# An Integrative Review of Specialised Nursing Career Frameworks to Develop a Nursing Career Framework for Registered Nurses Working in Aged Care

**DOI:** 10.1111/jan.16674

**Published:** 2024-12-18

**Authors:** Sachini Thennakoon, Seng Giap Marcus Ang, Victoria Traynor, Karen Strickland

**Affiliations:** ^1^ School of Nursing and Midwifery Edith Cowan University Joondalup Western Australia Australia; ^2^ School of Nursing University of Wollongong Wollongong New South Wales Australia; ^3^ School of Clinical Sciences, Faculty of Health and Environmental Sciences Auckland University of Technology Auckland New Zealand; ^4^ School of Nursing, Midwifery & Public Health University of Canberra Canberra Australian Capital Territory Australia; ^5^ School of Nursing, Midwifery and Paramedic Practice Robert Gordon University Aberdeen UK

**Keywords:** aged care, career framework, career pathway, career progression, competency framework, integrative review, nursing, nursing homes, professional development, retention

## Abstract

**Aim:**

The aim of this study is to synthesise literature on specialised nursing career frameworks to inform the development of an aged care nursing career framework.

**Design:**

An integrative review was conducted.

**Method:**

The review followed Whittemore and Knafl's five‐step integrative review method. To appraise the quality of the studies, the Quality Appraisal for Diverse Studies tool for peer‐reviewed articles and the ACCODS (Authority, Accuracy, Coverage, Objectivity, Date, and Significance) checklist for grey literature were used. Data were extracted and synthesised using the constant comparison method.

**Data Source:**

The electronic databases of CINAHL, Medline, PsycINFO and Google Scholar were searched to identify peer‐reviewed articles and grey literature reporting on specialised nursing career frameworks for registered nurses.

**Results:**

Eight studies were reviewed, and the findings were presented corresponding to each of the three research questions of the review. First, the research methods adopted to develop specialised nursing career frameworks were described based on the research design, stakeholder involvement and data collection methods. Second, the key elements of specialised nursing career frameworks were identified as career pathways, nursing competencies and roles and progression between the career levels. Third, the findings suggested that the key purposes for developing specialised nursing career frameworks are to improve professional development, recruitment and retention and to promote consistency and quality in nursing practice.

**Conclusion:**

The study highlights a significant gap in the evidence base of career frameworks for registered nurses in aged care, emphasising the need for future research. This review answered three research questions: methods for developing specialised nursing career frameworks, their key components and main purposes, providing insights to guide healthcare organisations and researchers. The findings indicate that career frameworks are primarily intended to promote knowledge and skills development and may also to bolster recruitment and retention rates, and support nurses' career advancement, but there is limited evidence on implementation, evaluation and sustainability.

**Implications for the Profession:**

The findings will guide healthcare organisations and future researchers with methods and techniques to develop specialised nursing career frameworks. Implementing a specialised nursing career framework in aged care could enhance continuous professional development, recruitment, retention and career progression among nurses.

**Impact:**

Nursing career frameworks have been widely applied to address professional development and retention objectives. However, there is limited evidence available to formulate a career framework for registered nurses working in aged care. The review identified the research methods adopted to develop specialised nursing career frameworks along with the key elements of specialised nursing career frameworks. These findings will guide employers and future researchers in developing evidence‐based aged‐care nursing career frameworks. Additionally, the findings will guide registered nurses in using career frameworks as a tool to facilitate career advancement and competency development. Furthermore, the review recognised the key purposes for developing specialised nursing career frameworks, suggesting that the meaningful adoption of career frameworks could be utilised as a strategic approach for enhancing retention and workforce development of the aged care workforce.

**Reporting Method:**

The Preferred Reporting Items for Systematic Reviews and Meta‐Analyses (PRISMA) guideline was used for reporting.

**Patient or Public Contribution:**

No patient or public contribution.

**Trial Registration:**

The integrative review protocol was registered in the PROSPERO international prospective register of systematic reviews database (registration no: CRD42022354728)

**Summary**

- Career frameworks are often recommended as a professional development strategy in nursing, but limited evidence exists to guide the development of specialised nursing career frameworks.
- This review adds a comprehensive overview of the research methods and techniques that can be adopted to develop specialised nursing career frameworks and their key elements, which can guide healthcare organisations and future researchers in developing specialised nursing career frameworks.
- The review identified a lack of evidence‐based nursing career frameworks for nurses working in aged care globally, highlighting a significant gap in the literature which offers the foundation for future researchers to develop specialised nursing career frameworks for aged care nurses.

## 1 Introduction

The global population is rapidly ageing, with over one billion people aged over 65, and there is an expectation that it will double by 2050 (World Health Organization 2022). Advanced age is associated with a range of physical, psychosocial and neurological impairments, causing higher prevalence rates of multimorbidities, frailty and functional disability among older individuals (Purfarzad et al. 2019). These complex health conditions intensify the healthcare care needs of older people and increase their likelihood of experiencing disabilities and requiring support from residential aged care facilities (RACFs) (Backhaus et al. 2015; Phelan and McCormack 2016; Vermeerbergen, Van Hootegem, and Benders 2017).

In RACFs, providing high‐quality care requires an adequate number of nursing professionals with specialised knowledge, skills and compassion that can address the unique needs of older people (Phelan and McCormack 2016). However, globally, there is a significant challenge in attracting and retaining registered nurses in the aged care sector (Backhaus et al. 2015; Moloney et al. 2017; Resnick et al. 2022). For instance, in Australia, the percentage of registered nurses working in aged care has declined significantly, with reports indicating a shortage of 41% of registered nurses working in aged care (Royal Commission into aged Care Quality and Safety 2021). In the United Kingdom, the turnover rates among nurses were reported to be 41% (Fitzpatrick et al. 2022) and Lee (2021) reported 1‐year turnover rates of aged care nurses were between 19% and 55%.

Several reasons contribute to the nurses' shortages in RACFs, including low staffing levels, lower pay, burnout and a lack of professional identity, discouraging nurses from a career in aged care (Hodgkin et al. 2016). The lack of career pathways and professional development opportunities is recognised as a major contributing factor to the shortage of registered nurses working in aged care (Fitzpatrick et al. 2022; Hodgkin et al. 2016). Without clear career pathways, nurses receive limited support and guidance for career progression, skills advancement and continuous professional development, leading to demotivation among nurses to continue their careers in aged care (Department of Health and Aged Care 2018; Karantzas et al. 2012; Moloney et al. 2017; Resnick et al. 2022). Consequently, nurses often shift to career options in other nursing specialities where professional development and career progression are more promising (Moloney et al. 2017). Moreover, aged care nursing is often portrayed as a less attractive career option, with limited opportunities for career progression and skills advancement, further exacerbating the challenge to attract registered nurses into aged care (Royal Commission into aged Care Quality and Safety 2021).

Globally, gerontological nursing competency frameworks exist to support and guide the competency development of registered nurses (Chunlan et al. 2020; Dijkman et al. 2022; Hayes and Naughton 2022; Traynor et al. 2024). However, there is a significant limitation in the international literature regarding the professional development programmes that have dedicated career planning components and career pathways (Fitzpatrick et al. 2022). For example, within Australia, national‐level inquiries such as the Royal Commission on Aged Care Quality and Safety (RCACQS) and the Aged Care Workforce Strategy Taskforce have highlighted a significant deficiency in nationally recognised career progression pathways (Australian Government Department of Health and Aged Care 2018; Royal Commission into aged Care Quality and Safety 2021). Recommendations have been made to establish nationally recognised career frameworks as a potential strategy to address career advancement and to bolster the recruitment and retention rates of registered nurses (Australian Government Department of Health and Aged Care 2018; Fitzpatrick et al. 2022; Royal Commission into aged Care Quality and Safety 2021).

Career frameworks have been frequently advocated as a professional development strategy in nursing as they serve to provide a roadmap that helps to guide employees in their career progression and provide structured guidance in skills and professional advancement that are required (Intensive Care NSW 2023; Ministry of Health and District Health Boards New Zealand 2007; Li et al. 2022; Nashwan 2023; Pertiwi and Hariyati 2019). Career frameworks outline clear career pathways that help nurses to visualise their long‐term career goals, strategically plan and navigate career trajectories, identify necessary steps and make informed decisions about their professional development (Bernard and Oster 2018).

Although career frameworks have been widely applied across a wide range of nursing specialities, there is limited evidence to inform the development of specialised nursing career frameworks. Previous research primarily focused on developing nursing competency frameworks, providing insights into the development of nursing competencies (Batt, Tavares, and Williams 2019). However, there is a significant limitation in the studies that report information on the nursing career frameworks, including the key features, attributes, research methods and theoretical frameworks which can inform the development of nursing career frameworks. A preliminary search on Medline did not yield any reviews conducted on synthesising evidence related to the development of specialised nursing career frameworks. Therefore, this integrative review was conducted to identify research related to the development of a specialised nursing career framework as part of a larger study to inform the development of an aged care nursing career framework in Australia.

## 2 Aims and Research Questions

This integrative review aimed to systematically synthesise studies reporting on the development of specialised nursing career frameworks. In this review, ‘specialised nursing career framework’ refers to career frameworks designed for nurses providing care for patients in a specific discipline or area.

The research questions are

1. What are the research methods adopted to develop specialised nursing career frameworks?
2. What are the key features of specialised nursing career frameworks?
3. What are the purposes of developing specialised nursing career frameworks?

## 3 Methods

### 3.1 Design

An integrative review was conducted to synthesise international research evidence related to developing specialised nursing career frameworks. The integrative review method combines diverse research designs, including empirical and theoretical research, to provide a comprehensive understanding of a phenomenon of interest (Oermann and Knafl 2021). The choice of the integrative review method was appropriate in this regard, considering the limited evidence and methodological heterogeneity of available studies to answer the three research questions (Da Silva, Brandão, and Ferreira 2020; Munn et al. 2018). Besides mapping the type of framework, key features and characteristics of the study design, the included studies were also critically appraised to determine the research quality and rigour. The review followed five steps described by Whittemore and Knafl (2005), which include (1) problem identification, (2) literature search, (3) data evaluation, (4) data synthesis and (5) presentation. The review adhered to the Preferred Reporting Items for Systematic Reviews and Meta‐Analyses (PRISMA) guidelines for reporting (Page et al. 2020).

### 3.2 Search Strategy

A systematic search was conducted in four electronic databases: CINAHL, MEDLINE, PsycInfo and Google Scholar. The keywords used were ‘nurs*’ and ‘career frameworks’ combined with boolean operators AND and OR. The full search strategy is provided in Appendix 1, Table A1. Additionally, a manual search of Google and the reference lists of eligible studies were searched to identify any missed articles. The search was completed in June 2023.

### 3.3 Inclusion and Exclusion Criteria

The review included peer‐reviewed research and grey literature published in English between 2000 and 2023, focusing on the development of specialised nursing career frameworks for registered nurses in any clinical care. Studies related to the development of career frameworks for registered nursing students, reviews, editorials and commentaries were excluded. Since the review focused on clinical nursing career frameworks, studies on non‐clinical nursing career frameworks, such as research and academic nursing career frameworks, were excluded.

### 3.4 Screening

**FIGURE A1:**
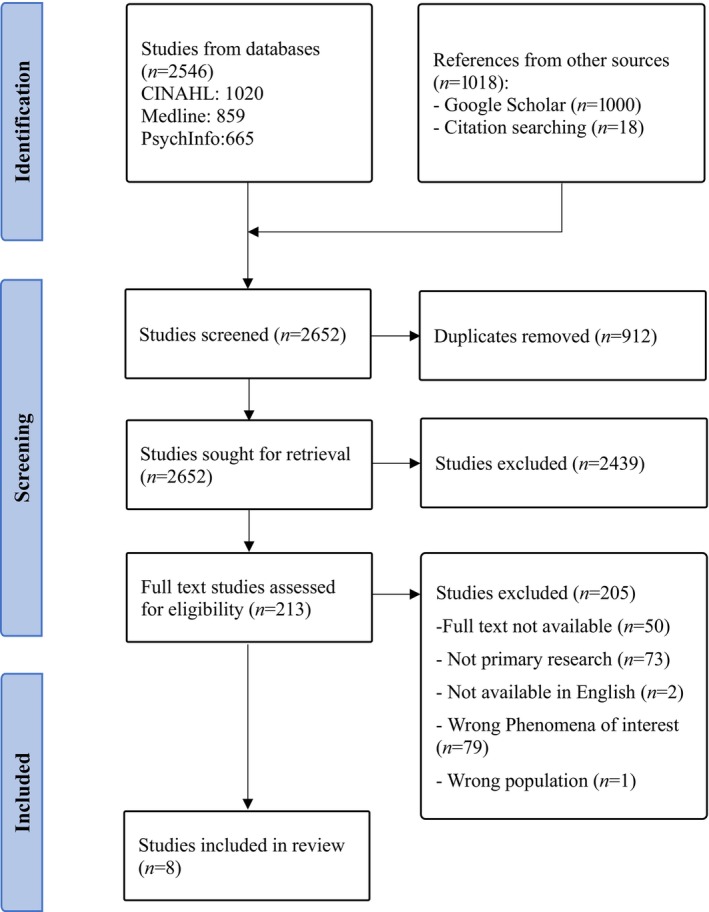
PRISMA flow chart.

The search yielded a total of 3564 articles (Appendix 6). Search results were then exported to COVIDENCE for data screening. After removing 912 duplicated studies, 2652 articles remained for screening by title and abstract. Following the exclusion of 2439 references, 213 articles were reviewed in full text against the inclusion and exclusion criteria, whereby 205 were excluded. Finally, eight studies, including five peer‐reviewed journal articles and three sources from grey literature, were considered for data extraction. Appendix 5, Figure A1 shows the PRISMA flow chart for the screening process. Two study team members independently screened the studies at all stages, whereby the conflicts were resolved through discussions with the research team at the end of each screening stage.

### 3.5 Quality Appraisal

The quality of peer‐reviewed journal articles was evaluated using the Quality Assessment with Diverse Studies (QuADS) tool (Harrison et al. 2021). The QuASD tool, as detailed in Appendix 2, Table A2, comprises 13 quality assessment criteria appraising the quality related to theoretical or conceptual underpinning, research aims, research setting and target population, research design, data collection and analysing methods, recruitment, evidence of the research stakeholders and availability of a critical discussion on strengths and limitations. A four‐point Likert scale (0 = Not mentioned, 1 = Very slightly, 2 = Moderately and 3 = Completely) was used to rate each quality criterion with a maximum score of 42 points. In this review, the final score of each article was calculated against the maximum score of 42 and converted to a percentage for comparison purposes. The ACCODS (Authority, Accuracy, Coverage, Objectivity, Date, and Significance) checklist introduced by Tyndall (2010) was used to evaluate the quality of grey literature. Using this tool, the quality of the included studies was rated by answering ‘yes’, ‘no’ or ‘maybe’ for the quality assessment questions under each quality criterion. Two study team members were involved in the quality appraisal, and any conflicts were resolved through discussions with other study team members.

The review included eight studies, five peer‐reviewed publications (Davis et al. 2007; Holloway 2012; Howard 2016; Tucci, McClain, and Peyton 2022; Wolf et al. 2012) and three grey literature sources (Australian Primary Health Care Nurses Association [APHCNA] 2018; Health Education England 2020; Royal College of Nursing [RCN] 2022). The peer‐reviewed publications had an average to moderate quality, with scores ranging from 43% to 83%, except for one study that scored only 26% due to a lack of description of the study methods (Wolf et al. 2012). The included grey literature sources demonstrated high quality across the five criteria assessed, with all studies receiving a rating of 4 out of 5, with the lacking component being details about theoretical frameworks used to inform the study reported. The outcome of the quality appraisal of grey literature is provided in Appendix 3, Table A3. None of the studies were excluded based on the quality of the articles due to the limited evidence‐based research describing nursing career framework development.

### 3.6 Data Extraction and Synthesis

A standardised data extraction form was developed after a discussion with the review team. The data extraction table is provided in Appendix 4, Table A4. Data were extracted using the following details: author name, year of publication, aim, research design, data collection, participants and key findings. Data synthesis followed the constant comparison method suggested by Whittemore and Knafl (2005). This method encompassed five steps: data reduction, data display, data comparison, conclusion, verification and presentation. As the first step, a predefined classification system was used to categorise and reduce the data into subgroups. In this review, the research questions were used as the classification system for data reduction, and the data were classified into three subgroups. Following that, the data were coded and displayed using tables, enabling comparison between the primary resources. The codes were thoroughly examined to identify the patterns, and data‐driven subcategories and categories were developed by clustering similar data. During each step, data displays were compared within categories and between primary resources regardless of the research design of the included studies. An iterative process was conducted during this process. The final set of categories corresponding to each research question was finalised with the agreement of the research team and further verified with the primary sources for accuracy before presenting. The findings were presented under the three research questions of this review. NVivo software version 1.6 was used for this data analysis.

## 4 Results

### 4.1 Characteristics of the Included Studies

Of the eight included studies, five were peer‐reviewed journal articles (Davis et al. 2007; Holloway 2012; Howard 2016; Tucci, McClain, and Peyton 2022; Wolf et al. 2012), and three were grey literature sources (APHCNA 2018; Health Education England 2020; RCN 2022). Three studies were conducted in the United Kingdom (Davis et al. 2007; Health Education England 2020; RCN 2022), two from New Zealand (Holloway 2012; Howard 2016) and one study from Africa (Wolf et al. 2012), Australia (APHCNA 2018) and the United States respectively (Tucci, McClain, and Peyton 2022). The reviewed career frameworks belonged to different nursing specialities, including cancer nursing (RCN 2022), primary care nursing (APHCNA 2018), emergency nursing (Wolf et al. 2012), specialised nursing (Howard 2016), occupational health nursing (Holloway 2012), mental health nursing (Health Education England 2020) and diabetes nursing (Davis et al. 2007). However, this review did not find career frameworks for gerontological nursing and the aged care sector.

### 4.2 Main Findings

The study findings were presented corresponding to the research questions that guide this review: (1) the research methods adopted to develop specialised nursing career frameworks, (2) the key elements of specialised nursing career frameworks and (3) the key purposes of developing nursing career frameworks.

#### 4.2.1 The Research Methods Adopted to Develop Specialised Nursing Career Frameworks

The research methods for developing specialised nursing career frameworks were presented within three categories (1) research design, (2) stakeholder involvement and (3) data collection methods.

##### 4.2.1.1 Research Design

Four reviewed studies adopted qualitative research designs in developing their nursing career frameworks (Davis et al. 2007; Holloway 2012; Howard 2016; Wolf et al. 2012). The study designs varied slightly from each other: Howard (2016) followed qualitative participatory action research, aiming that collaborative problem‐solving processes between participants would link practice, theory and social change. Holloway (2012) used a qualitative descriptive multi‐method inquiry approach to develop a comprehensive nursing career framework. Davis et al. (2007) and Wolf et al. (2012) reported adopting qualitative research design without specifying the methodological details. The remaining studies did not specify the research design that guided the career framework development (APHCNA 2018; Health Education England 2020; RCN 2022; Tucci, McClain, and Peyton 2022).

##### 4.2.1.2 Stakeholder Involvement

Stakeholder involvement ensures the accurate representation of diverse needs and perspectives of all relevant key informants (Lepre et al. 2021). Seven of eight studies included stakeholders such as healthcare professionals, patients and consumers for the development of the nursing career framework. Six studies included healthcare professionals including nurses who have different levels of experiences from novice to expert (APHCNA 2018; Davis et al. 2007; Health Education England 2020; Holloway 2012; Howard 2016; RCN 2022; Wolf et al. 2012), nurse researchers and academics (APHCNA 2018; Holloway 2012; Wolf et al. 2012), policymakers (APHCNA 2018; Holloway 2012) and healthcare professionals from healthcare peak bodies (APHCNA 2018; Davis et al. 2007; Holloway 2012; RCN 2022). One study engaged patients and consumers besides healthcare professionals (Davis et al. 2007). Three studies recruited stakeholders through purposive sampling or snowballing methods (APHCNA 2018; Davis et al. 2007; Howard 2016). However, the remaining five studies did not report their sampling technique (Health Education England 2020; Holloway 2012; RCN 2022; Tucci, McClain, and Peyton 2022; Wolf et al. 2012). Stakeholders assumed varied key roles in developing nursing career frameworks, encompassing steering projects, identifying practice needs and key elements of career frameworks, reviewing frameworks, as well as providing advisory input.

##### 4.2.1.3 Data Collection Methods

Various data collection methods were used to develop nursing career frameworks. Literature reviews, workshops and interviews were commonly conducted to identify practice needs and key elements of specialised nursing career frameworks. Five studies used literature reviews, which entailed document analysis (Holloway 2012), rapid review (RCN 2022), desk data analysis (Tucci, McClain, and Peyton 2022) and comprehensive literature searches (APHCNA 2018; Howard 2016). These literature searches explored existing nursing career frameworks, policies, nursing theories and legislations to identify the structure and key components encompassing nursing career frameworks (APHCNA 2018; Holloway 2012; Howard 2016; Tucci, McClain, and Peyton 2022). Three studies conducted key stakeholder workshops to identify practice needs (Davis et al. 2007; Wolf et al. 2012) and key elements of career frameworks (APHCNA 2018; Davis et al. 2007; Wolf et al. 2012). Additionally, three studies utilised interviews with key stakeholders to gather information on key elements of specialised nursing career frameworks (APHCNA 2018; Holloway 2012; Tucci, McClain, and Peyton 2022). These interview modes varied, including telephone interviews (Tucci, McClain, and Peyton 2022) and face‐to‐face interviews (Holloway 2012).

Surveys and consensus development methods were utilised at the last stages of nursing career framework development studies. APHCNA (2018) used an online survey to gather consumer feedback on the functionality of the nursing career framework. Howard (2016) conducted two surveys retrospectively and prospectively to assess participants' perspectives on the study and their role in participatory action research. The consensus development method was used in three studies, including Delphi (APHCNA 2018; Davis et al. 2007; Holloway 2012) and nominal group technique (Davis et al. 2007), to obtain expert agreement on the key components of the nursing career frameworks (APHCNA 2018) and to establish an initial consensus of the specialised nursing career frameworks (Holloway 2012). However, only one study provided explicit details on the consensus achievement process (Holloway 2012).

#### 4.2.2 Key Elements of Specialised Nursing Career Frameworks

Key elements of specialised nursing career frameworks were identified within three main categories (1) career pathway, (2) nursing competencies and role and (3) progression between the career levels.

##### 4.2.2.1 Career Pathway

Career pathways support a progressive continuum of practice, aiding nurses in navigating their career trajectories towards higher levels of practice. All the nursing career frameworks included a structured career pathway that offered vertical career advancement (APHCNA 2018; Davis et al. 2007; Health Education England 2020; Holloway 2012; Howard 2016; RCN 2022; Tucci, McClain, and Peyton 2022; Wolf et al. 2012). A career pathway can encompass different practice levels, spanning from beginner to expert levels. The number of practice levels of the reviewed career frameworks ranged from three to five, with different terminology used for each practice level. For instance, APHCNA (2018) introduced a primary care career pathway with three practice levels: foundation, intermediate and advanced. Davis et al. (2007) outlined a diabetes nursing career pathway with four levels of practice ranging from competent to consultant level, and Wolf et al.'s (2012) emergency nursing career pathway was consistent with Benner's (1984) earlier work, with five practice levels from novice to expert. Despite variations in the number of practice levels and the terminology used, aspects related to the degree of complexity, responsibility, expertise and decision‐making were shown to increase as nurses progressed towards a higher level of practice.

##### 4.2.2.2 Nursing Competencies and Nurses' Role

Six reviewed nursing career frameworks were primarily competency‐based career frameworks, delineating requisite competencies at different practice levels (Davis et al. 2007; Health Education England 2020; Holloway 2012; Howard 2016; RCN 2022; Wolf et al. 2012). This approach offered a clear understanding of the knowledge, skills and proficiencies pertinent to the respective level of practice for providing safe, quality patient care. Furthermore, competencies outlined across diverse practice levels aid nurses in identifying areas necessitating competency advancement to achieve career progression. Although the nursing competencies were tailored to different nursing specialities and contexts, competency advancement along the career pathways shared similar patterns and features across studies.

Only two studies incorporated nursing roles within their career frameworks (APHCNA 2018; Wolf et al. 2012). The APHCNA (2018) adopted a role‐based approach that outlined the scope of primary care nurses across three levels of expertise, which aligned with a list of activities that registered nurses are required to perform to meet the potential breadth of the role. Similarly, Wolf et al. (2012) incorporated nurse practice roles corresponding to different practice levels, along with other key elements.

##### 4.2.2.3 Progression Between the Practice Levels

Facilitating career progression is a key attribute of a nursing career framework, warranting an essential component in a career framework. However, only three studies outlined the key determinants of career progression within the nursing career framework (Health Education England 2020; Tucci, McClain, and Peyton 2022; Wolf et al. 2012). These studies underscored educational qualifications (Health Education England 2020; Tucci, McClain, and Peyton 2022), work experience (Tucci, McClain, and Peyton 2022) or cognitive understanding of patient care needs (Wolf et al. 2012) as key determinants governing career progression. Health Education England (2020) stipulated minimum educational qualifications that start with a first degree in mental health nursing and the minimum of a master's degree to reach the highest practice level, along with workplace‐based training. Tucci, McClain, and Peyton (2022) outlined that nurses must attain the requisite education in conjunction with the defined years of experience to ascend in the career pathway, whereas Wolf et al. (2012) asserted cognitive understanding of patient care needs and critical thinking as key determinants for career progression, although education, experience and psychomotor skills were not excluded.

Wolf et al. (2012) advocated using Lasater's clinical judgement rubric to assess nurses' cognitive understanding of patient care situations and determine their career progression based on the outcome of their cognitive thinking assessment. This process allows nurses to advance in their career pathways when they demonstrate increased expertise and clinical judgement.

#### 4.2.3 Purposes for Developing Specialised Nursing Career Frameworks

This explores the key purposes of developing nursing career frameworks. Three categories emerged in this context: (1) professional development, (2) recruitment and retention and (3) promoting consistency and quality in nursing practice.

##### 4.2.3.1 Professional Development

All included studies highlighted supporting continuous professional development as a key focus for the development of a nursing career framework (APHCNA 2018; Davis et al. 2007; Health Education England 2020; Holloway 2012; Howard 2016; RCN 2022; Tucci, McClain, and Peyton 2022; Wolf et al. 2012). These career frameworks offer a structured career progression map, aiding nurses in identifying career progression opportunities and establishing individual career advancement goals (RCN 2022; Wolf et al. 2012). Furthermore, career frameworks serve as a self‐assessment tool enabling nurses to reflect on their current knowledge, skills and proficiencies against the benchmarks delineated within nursing career frameworks (APHCNA 2018; Health Education England 2020; RCN 2022). This reflective process aids in identifying knowledge gaps and guiding the pursuit of education and training to foster professional development (RCN 2022). Notably, career frameworks not only benefit employees but also can assist employers and service providers by providing accurate data to establish and negotiate structured and transparent career pathways for nurses (Howard 2016; RCN 2022).

Promoting continuous education stands out as a major goal of developing nursing career frameworks across all the studies (APHCNA 2018; Davis et al. 2007; Health Education England 2020; Holloway 2012; Howard 2016; RCN 2022; Tucci, McClain, and Peyton 2022; Wolf et al. 2012). RCN (2022) and Wolf et al. (2012) highlighted that career frameworks serve as a mapping tool to identify the proficiencies that correlate with different nursing practice levels, in turn facilitating employers, higher education institutes and policymakers in designing educational and training programmes. Furthermore, career frameworks offer a reference point to evaluate existing undergraduate and postgraduate curricula, driving updates and redesigning nursing curricula based on the knowledge and competency development needs of the healthcare context (APHCNA 2018; RCN 2022; Wolf et al. 2012).

##### 4.2.3.2 Recruitment and Retention

Six studies posited career frameworks as a key workforce recruitment and retention strategy (APHCNA 2018; Health Education England 2020; Holloway 2012; Howard 2016; RCN 2022; Wolf et al. 2012). Career frameworks have the potential to recruit nurses by upgrading the professional image of nursing and reengineering less attractive nursing specialities as rewarding and fulfilling career options with multiple career progression opportunities, which in turn can recruit individuals who might not have considered these nursing specialities as a career option (APHCNA 2018; Health Education England; Wolf et al. 2012). These studies also reported that career frameworks could enhance workforce retention as supporting the professional development of nurses can significantly influence retention (APHCNA 2018; Holloway 2012; RCN 2022).

##### 4.2.3.3 Promoting Consistency and Quality in Nursing Practice

Four studies reported that career frameworks were developed to minimise perceived variations in education, competencies, scope of practice, roles and titles across different nursing organisations, settings and regions (APHCNA 2018; Health Education England 2020; RCN 2022; Wolf et al. 2012). This, in turn, fosters consistency and uniformity in levels of knowledge, skills, competencies and education required for career progression across diverse settings. Moreover, nursing career frameworks also guide establishing standards and performance indicators to evaluate staff development (Holloway 2012; RCN 2022). Career frameworks can also be featured in job applications, audits and ongoing quality improvement activities (Holloway 2012). Additionally, the APHCNA (2018) and the RCN (2022) suggested integrating career frameworks into job applications, position descriptions, performance appraisals and succession planning to ensure alignment with the nursing speciality standards.

## 5 Discussion

The primary aim of this integrative review was to systematically synthesise studies reporting on the development of specialised nursing career frameworks. The research team is using the findings from the review to inform the development of a new nursing career framework for registered nurses working in Australian aged care. This review included three grey literature and five peer‐reviewed research studies. The findings were presented corresponding to the three main research questions of this study. Three categories emerged to answer the first research question on research methods adopted to develop specialised nursing career frameworks: research design, stakeholder involvement and data collection methods. Second, the key elements of specialised nursing career frameworks were identified as career pathways, nursing competencies and roles and progression between the career levels. Third, the purposes for developing specialised nursing career frameworks emerged as supporting professional development, recruitment and retention and promoting consistency and quality in nursing practice.

Most articles reviewed were published by professional nursing organisations and the methodological quality, such as research designs, sampling criteria and data collection methods for developing the nursing career framework, was poorly described. However, this review did not exclude any studies due to limited details on the research methodology, as the primary purpose of this review is to provide an extensive discussion of the specialised nursing career framework available in the international literature.

This review identified a global absence of an evidence‐based nursing career framework for nurses working in gerontological nursing and aged care. This observation aligns with a recent literature review conducted by Fitzpatrick et al. (2022), which reported a significant limitation in the professional development programmes for nurses employed in aged care, particularly career pathways and career planning. This knowledge gap calls for future research in developing a specialised career framework for aged care nurses to address the ongoing inconsistencies in career planning, workforce recruitment, retention and competency advancement of this group of specialist nurses. The research team will lead this work in Australia.

This review has identified a diverse range of research techniques and approaches used to develop the nursing career frameworks. Healthcare professionals were involved as key stakeholders in developing the nursing career frameworks in all included studies. Incorporating healthcare professionals facilitates an accurate representation of practice needs, leading to the development of comprehensive career frameworks (Lepre et al. 2021). The specialised nursing career frameworks were mainly developed by nurses for nurses, allowing an accurate representation of the diverse experiences and expertise found within the nursing profession, thus enhancing its applicability to a wider practitioner population. Some studies clearly indicated the level of experience of nurses involved in developing the career frameworks, noting the inclusion of both novice and expert nurses. However, the other studies primarily focused on experts in the healthcare context. It is crucial to integrate the views and opinions of the nurses working in the clinical practice who are the end users of such career frameworks therefore involving a balanced representation of both novice and expert nurses may ensure the career frameworks are effective and relevant to all practice levels.

Most studies adopted a qualitative research method for developing the nursing career frameworks because it can generate rich and contextually relevant findings to inform the development of the career frameworks (Blustein et al. 2005). However, adopting only qualitative research methods can limit the number of participants that can be involved in developing the career framework. For instance, two studies that used the qualitative research method had a sample size of less than twenty participants. The smaller sample size can hinder the generalisability and applicability of the framework across different locations and levels of practice due to the limited representation of the key informants (Creswell and Clark 2017). In contrast, adopting quantitative research might improve the generalisability of the frameworks; however, relying on only quantitative research may not facilitate a deep exploration of participants' voices, potentially missing out on nuanced and context‐specific insights (Creswell and Clark 2017). Hence, a balanced approach that includes both qualitative and quantitative research is suggested to improve the comprehensiveness and generalisability of the nursing career framework through triangulation of the study findings.

Career pathways were identified as the foundation of nursing career frameworks, with other key elements aligned to correlate to different practice levels of a career pathway. The other key elements of the nursing career frameworks identified during this review were nursing competencies, nursing roles and prerequisite qualifications for career advancement. It is crucial to note that the specific key elements found in this review were diverse across the studies. For instance, six career frameworks exclusively incorporated nursing competencies, whereas other frameworks encompassed nursing roles or a combination of both. The selection of key elements depending on the study aims and context ensures that the nursing career frameworks remain responsive to the evolving demands of the profession, fostering relevance and effectiveness of the nursing career frameworks.

All nursing career frameworks included in this review have a clear purpose of supporting the professional development of nurses and career progression. Career progression is facilitated by outlining a career pathway that enables nurses to envision long‐term career goals, make informed decisions and achieve the necessary qualifications for career advancement (Nashwan 2023). To support career progression, the career framework must identify the necessary qualifications, prerequisites and mechanisms of the career advancement process (Moore, Meucci, and McGrath 2019). However, only three reviewed studies explicitly outlined key determinants of career advancement, such as education qualification, clinical experience and nurses' cognitive understanding of patient care needs. It is important to note that the inclusion of all three criteria for career advancement facilitates an effective career progression, as relying solely on education without adequate experience might not improve an individual's expertise, and possessing both education and experience might not fully equate to the clinical decision‐making and critical thinking ability required for career advancement (Oyetunde and Oluwafunke 2015; Moore, Meucci, and McGrath 2019). Therefore, future studies are encouraged to comprehensively outline the key determinants for career advancement to ensure an effective and meaningful career advancement process.

Most of the included career frameworks demonstrated a common purpose of supporting nursing competency development. This aligns with existing evidence of professional development models such as career frameworks, career ladders and career pathways, which often support the competency advancement of nurses (Oyetunde and Oluwafunke 2015; Pertiwi and Hariyati 2019). A literature review that aimed to explore the impact of the career ladder system on nurses' job satisfaction found an association between implementing a career ladder programme and increased nurses' skills and competencies (Pertiwi and Hariyati 2019). Similarly, Naughton et al. (2023) also identified a statistically significant improvement in nursing competencies after participating in a career pathway programme. Li et al. (2022) found that participation in clinical ladder programmes improved nurses' professional competence, safety and quality of care. It was hypothesised that the career framework supports competency advancement by outlining the nursing competencies corresponding to different practice levels and serves as a self‐assessment tool for nurses to identify the knowledge gaps and development needs for career advancement (RCN 2022).

This review identified that most nursing career frameworks were developed as a strategic approach to improve the recruitment and retention of nurses. Globally, nursing career frameworks have been applied as a successful workforce recruitment and retention strategy over the last few decades (Li et al. 2022; Pertiwi and Hariyati 2019; Oyetunde and Oluwafunke 2015; Nashwan 2023). For instance, it was further identified that the implementation of a career ladder contributed to an 85% retention rate among the nurses working in a large hospital network. Furthermore, evidence also suggested that the inclusion of a career framework resulted in a significant decline in nursing turnover rates (Brook et al. 2019). Professional development, often accompanied by the implementation of a nursing career framework, plays a major role in determining an employee's career trajectory, which leads to increased job satisfaction, engagement and embeddedness among nurses, ultimately leading to workforce retention (Bernard and Oster 2018; Pertiwi and Hariyati 2019). Implementing a career framework may potentially enhance the professional image of less attractive nursing specialities, such as aged care nursing, by presenting career advancement and training opportunities (Naughton et al. 2023).

Although the review findings indicated that career frameworks have a positive impact on the workforce development of nurses, none of the studies have assessed their impact on implementation in the clinical setting, creating a gap in the measurable impact of career frameworks. This calls for future research in evaluating the effectiveness of career frameworks on workforce development among nurses. It is also important to note that career frameworks are not standalone solutions, as their impacts are influenced by the organisational contexts and constraints of the health system. This requires not only the establishment of career frameworks but also meaningful integration of career frameworks within the organisational system and the contribution of employers in implementing the career frameworks to support the employees' progression through career frameworks. Otherwise, poorly implemented career frameworks may negatively impact employees by creating dissatisfaction when the promised career progression or rewards cannot be materialised.

### 5.1 Strengths and Limitations

This review followed a systematic process of searching, extracting and synthesising the study findings. Two independent reviewers were involved in the screening process, and data analysis and interpretation were conducted with the consensus of the research team, mitigating researcher bias. Adopting the integrative review method is a strength because it allows for the inclusion of diverse research designs and grey literature sources that discuss the development of a nursing career framework. However, the review excluded studies that did not describe the development of a nursing career framework and only included the four most pertinent databases for the search. The review also excluded nursing career frameworks related to non‐clinical nursing career frameworks and restricted to articles published in English, thus subjecting to potential selection bias.

### 5.2 Implications for Practice and Research

The review suggests that the implementation of a nursing career framework could promote the professional development of nurses and improve workforce retention and overall job satisfaction. However, none of the studies have informed the measurable impact of the nursing career frameworks in achieving such goals, highlighting the need for future research.

The findings offer future researchers and healthcare organisations a comprehensive outline of the key components of a nursing career framework and the research methods that can be utilised to develop future nursing career frameworks. Although the review provided an overview of the importance and strategies to develop a nursing career framework, it has identified a significant knowledge gap globally regarding a nursing career framework for the aged care context. Future studies on developing a career framework will benefit from tailoring these findings to respective study settings, contexts, regions and study aims for the best outcomes.

## 6 Conclusion

This integrative review synthesised evidence related to the development of specialised nursing career frameworks. By appraising the evidence on career frameworks, the foundation was laid for developing an aged care nursing career framework that can be tailored to the contexts of different countries. The review meticulously followed the established research methods, using the five‐step integrative review method of Whittemore and Knafl (2005) and adhering to the Preferred Reporting Items for Systematic Reviews and Meta‐Analyses (PRISMA) guideline. The findings were presented to indicate the research methods adopted to develop specialised nursing career frameworks, the key elements of specialised nursing career frameworks and the primary purposes for developing specialised nursing career frameworks. These findings provide valuable insights that will not only guide healthcare organisations but also inspire future researchers by offering methodologies and techniques to construct specialised nursing career frameworks. Most importantly, the implementation of a specialised nursing career framework within the aged care sector holds the potential to catalyse continuous professional development, bolster retention rates and provide clear pathways for career progression for nurses. Such frameworks have the potential to address the pressing shortage of registered nurses in the aged care workforce and contribute to the overall improvement of healthcare services provided to the ageing population when integrated and successfully implemented within the organisational system.

## Author Contributions

Made substantial contributions to conception and design, or acquisition of data, or analysis and interpretation of data: S.T., K.S., M.A., V.T.; Involved in drafting the manuscript or revising it critically for important intellectual content: S.T., K.S., M.A., V.T.; Given final approval of the version to be published. Each author should have participated sufficiently in the work to take public responsibility for appropriate portions of the content: S.T., K.S., M.A., V.T.; Agreed to be accountable for all aspects of the work in ensuring that questions related to the accuracy or integrity of any part of the work are appropriately investigated and resolved: S.T., K.S., M.A., V.T.

## Conflicts of Interest

The authors declare no conflicts of interest.

## Peer Review

The peer review history for this article is available at https://www.webofscience.com/api/gateway/wos/peer‐review/10.1111/jan.16674.

## Data Availability

The data that support the findings of this study are available from the corresponding author upon reasonable request.

## Appendix 1

**TABLE A1:** Search strategy.

Database	Search strategy
CINAHL	nurs*AND (“Career framework*” OR “Career path*” OR “Career map*”OR“Career advancement*”OR (MH “Clinical Ladder”) OR “Clinical ladder*”OR“Career progres*”)
MEDLINE	nurs*AND(“Career framework*”OR “Career path*”OR “Career map*”OR“Career advancement*”OR “Clinical ladder*” OR“Career progres*”OR“Clinical ladder*”OR“Career development”OR“Professional development”)
PsycINFO	nurs*AND(“Career framework*”OR “Career path*”OR“Career map*”OR “Career advancement”OR“Clinical ladder*”OR“Career ladder*”OR“Career progres*”)
Google Scholar	nurs*AND (“Career framework*” OR “Career path*”OR “Career map*”OR“Career advancement*”OR (MH “Clinical Ladder”) OR“Career progres*”)

## Appendix 2

**TABLE A2:** Methodological quality of peer reviewed articles.

Quality criteria	Tucci, McClain, and Peyton (2022)	Davis et al. (2007)	Holloway (2012)	Howard (2016)	Wolf et al. (2012)
(1) Theoretical or conceptual underpinning to the research	0	0	3	3	0
(2) Statement of research aim/s	3	2	3	3	2
(3) Clear description of research setting and target population	3	2	3	3	1
(4) The study design is appropriate to address the stated research aim/s	1	2	2	2	1
(5) Appropriate sampling to address the research aim/s	1	3	3	3	1
(6) Rationale for choice of data collection tool/s	1	1	3	2	1
(7) The format and content of the data collection tool is appropriate to address the stated research aim/s	2	3	3	3	1
(8) Description of data collection procedure	2	3	3	3	1
(9) Recruitment data provided	2	3	3	3	1
(10) Justification for analytic method selected	0	2	2	2	0
(11) The method of analysis was appropriate to answer the research aim/s	0	3	3	3	0
(12) Evidence that the research stakeholders have been considered in research design or conduct.	3	3	1	1	1
(13) Strengths and limitations critically discussed	0	3	3	3	0
Total score	18	30	35	34	10
Percentage	43%	71%	83%	81%	24%

## Appendix 3

**TABLE A3:** Methodological quality of grey literature.

	Health Education England (2020), United Kingdom	RCN (2022), United Kingdom	APHCNA (2018), Australia
Authority			
Individual author:			
Associated with a reputable organisation?			
Professional qualifications or considerable experience?			
Produced/published other work (grey/black) in the field?			
Recognised expert, identified in other sources?			
Cited by others? (use Google Scholar as a quick check)			
Higher degree student under “expert” supervision?			
Organisation or group:			
Is the organisation reputable? (e.g., W.H.O)	Yes	Yes	Yes
Is the organisation an authority in the field?	Yes	Yes	Yes
In all cases:			
Does the item have a detailed reference list or bibliography?	Yes	Yes	Yes
Accuracy			
Does the item have a clearly stated aim or brief?	Yes		Yes
Is so, is this met?	Yes		Yes
Does it have a stated methodology?	No	No	Yes
If so, is it adhered to?			Yes
Has it been peer‐reviewed?	Can't tell	Can't tell	Yes
Has it been edited by a reputable authority?	Yes	Yes	Yes
Supported by authoritative, documented references or credible	Yes	Yes	Yes
sources?	Yes	Yes	Yes
Is it representative of work in the field?	Yes	Yes	Yes
If no, is it a valid counterbalance?			
Is any data collection explicit and appropriate for the research?	Can't tell	Can't tell	Yes
If item is secondary material (e.g., a policy brief of a technical report)			
refer to			
the original. Is it an accurate, unbiased interpretation or analysis?			
Coverage			
Are any limits clearly stated?	No	No	No
Objectivity			
Opinion, expert or otherwise, is still opinion is the author's			
Standpoint clear?	Yes	Yes	Yes
Does the work seem to be balanced in presentation?	Yes	Yes	Yes
Date			
Does the item have a clearly stated date related to content?	Yes	Yes	Yes
If no date is given, but can be closely ascertained, is there a valid			
reason for its absence?			
Check the bibliography: have key contemporary material been	Yes	Yes	Yes
included?			
Significance			
Is the item meaningful? (This incorporates feasibility, utility and	Yes	Yes	Yes
relevance)	Yes	Yes	Yes
Does it add context?	Yes	Yes	Yes
Does it enrich or add something unique to the research?	Yes	Yes	Yes
Does it strengthen or refute a current position?	Yes	Yes	Yes
Would the research area be lesser without it?	Yes	Yes	Yes
Is it integral, representative, typical?	Yes	Yes	Yes
Does it have impact? (in the sense of influencing the work or	Yes	Yes	Yes
behaviour of others)	Yes	Yes	Yes
Does it have impact? (in the sense of influencing the work or behaviour of others)	Yes	Yes	Yes

## Appendix 4

**TABLE A4:** Summary of included studies.

Author, year, place	Aims	Research design	Participants	Data collection	Key findings
Holloway (2012), New Zealand	To develop a national specialist nursing framework	Qualitative descriptive and exploratory multi‐method enquiry	Twenty‐nine key informants and specialist nurses representing different nursing organisations were involved.	Modified e‐Delphi, semi‐structured interviews, literature reviews, document analysis and surveys were used for data collection	This study provides a capability framework for specialist nursing practice, which has practical implications to support the development of specialist nursing services, professional development and recognition pathways for nurse specialists
Wolf et al. (2012), Africa	To develop a career framework for emergency nursing practice in Africa	The framework was empirically tested using qualitative methodologies	The framework was developed by a group of international emergency nurses (academic and clinical nurses). The exact number of participants is not specified	The framework was developed through an international nurses' workgroup	This paper describes the challenges emergency nursing faces in Africa and the need for a framework. The framework includes 31 competencies and seven domains of nursing practice and encompasses five levels of expertise (Novice, advanced beginner, competent, proficient, expert). Benner's framework was used to determine the emergency nurses' level of expertise. This framework is intended to apply in nursing education, training and staffing
Davis et al. (2007), United Kingdom	To describe the development of an integrated career and competency framework for diabetes nursing	Qualitative research method	Over 200 nurses representing different sectors and grades in diabetes care were involved in this study	The study reports the use of value clarification exercises, workshops, Delphi and nominal group technique for data collection	Diabetes nursing competencies for a range of diabetes‐related areas were included for nurses at five levels of practice (Unregistered practitioners, competent nurses, experienced/proficient nurses, senior practitioners/expert nurses and consultant nurses). The framework has implications to inform nursing career pathways, support personal development planning and guide educational preparation
Howard (2016), New Zealand	To review and develop an integrated career and competency framework for occupational health nurses	The study used Qualitative participatory action research and developed the framework over six iterative phases	The study involved eight occupational health nurses in the participatory action group and involved a diverse group of stakeholders, but the exact number is not specified	The study utilised surveys, document analysis and participatory action research group meetings to develop the framework	The framework includes occupational health nursing skills and knowledge areas such as legislation and standards, health promotion, risk assessment, leadership and management skills, research, fitness for work and professionalism under three different practice levels: competent, proficient and expert. Implications include raising the profile of occupational health nurses, facilitate public health strategy and aid applying employment legislation in businesses
RCN (2022), United Kingdom	To provide a pathway for cancer nurses for career development and a framework for training, continuing development and education	Not specified	A diverse group of stakeholders, including an expert group of nursing professionals and peak bodies of cancer nursing, were involved, but the exact number is not mentioned	The study reports the use of a rapid literature review, mapping of existing competency frameworks and consultations for data collection	The career framework includes four levels of nursing practice (registration, enhanced, advanced or consultant practitioner levels) and cancer‐specific outcomes under pillars of professional practice. Framework provides a toolkit for various stakeholders to clarify cancer‐specific outcomes and improve nurses' education, training and professional development
APHCNA (2018), Australia	To develop a primary health care RN (Registered Nurse) Framework	Not specified	Over 1000 nursing professionals in primary healthcare nursing, non‐nursing and non‐primary healthcare experts were engaged to develop the framework	Delphi study, surveys, stakeholder interviews, stakeholder, workshops, briefings, prototype testing and interviews were used for data collection	The framework outlines the scope of practice for registered nurses (RNs) in primary health care across three levels of expertise (Foundation, Intermediate, Advanced) and five domains of practice: Clinical Care, Education, Research, Optimising Health Systems and Leadership. The purposes of the career framework include improving employment opportunities, building capacity, supporting skill transfer, strengthening nursing education and supporting nurses' recruitment and retention
Health Education (2020), England	Develop a framework for career progression in mental health nursing	Not specified	Expert reference groups representing key stakeholder organisations across the mental health sector were involved. The exact number of stakeholders is not mentioned	The study involved desk research and stakeholder events to develop the framework	The Framework includes five levels of practice from level 5–9, with characteristics and attributes ascribed to each level and core competencies required at each level of practice under four pillars of practice (clinical practice, education, facilitation of learning, leadership and development, research, evidence and development). The framework provides a tool for consistency in career development, helps in identifying competencies and aids in career planning and designing education programs
Tucci, McClain, and Peyton (2022), United States	This article describes the creation and implementation and outcomes of the clinical advancement program	Not specified	The study involved various stakeholders who are nurses, but the exact participant number is unclear	Literature reviews, solicited expert opinions, focus groups and phone interviews were used	The paper describes creating and implementing a clinical advancement program, known as the N‐CARE program, for oncology nurses at a comprehensive cancer centre. The program was developed to provide a structured career pathway for nurses, with defined performance expectations and requirements for promotion and role maintenance. The program includes a promotion process based on Benner's model of clinical competence, with nurses required to meet specific criteria and submit portfolios with exemplars of their clinical and leadership skills. The N‐CARE program has been successful in promoting nurses to higher levels of clinical competence, with a high pass rate for promotions to CN III and CN IV

## Appendix 6 Search Outcome for Electronic Databases

CINAHL searches.

**Table:**

	Query	Results
S10	S8 AND S9	1020
S9	S1 OR S2 OR S3 OR S4 OR S5 OR S6 OR S7	4479
S8	nurs*	1,031,358
S7	(MH “Clinical Ladder”)	993
S6	“Career progres*”	682
S5	“Clinical ladder*”	1074
S4	“Career advancement*”	796
S3	“Career map*”	21
S2	“Career path*”	2005
S1	“Career framework*”	85

Limiters—Publication Date: 20000101–20231231; English Language; Peer Reviewed; Language: English.

MEDLINE Searches.

**Table:**

	Query	Results
S14	S10 AND S13	859
S13	S1 OR S2 OR S3 OR S4 OR S5 OR S6 OR S7	5182
S12	S10 AND S11	11,733
S11	S1 OR S2 OR S3 OR S4 OR S5 OR S6 OR S7 OR S8 OR S9	39,424
S10	nurs*	1,167,206
S9	“career development”	18,477
S8	“professional development”	17,524
S7	“clinical ladder*”	206
S6	“career framework*”	42
S5	“career path*”	2710
S4	“career map*”	13
S3	“career advancement*”	1165
S2	“career ladder*”	326
S1	“career progres*”	909

Limiters—Peer Reviewed; Publication Date: 20000101–20231231; English Language.

Expanders—Apply equivalent subjects.

Search modes—Boolean/Phrase.

PsycINFO searches.

**Table:**

	Query	Results
S10	S8 AND S9	665
S9	S1 OR S2 OR S3 OR S4 OR S5 OR S6 OR S7	14,886
S8	nurs*	209,006
S7	“Career framework*”	52
S6	“Career path*”	2810
S5	“Career map*”	10
S4	“Career advancement*”	11,833
S3	“Clinical ladder*”	30
S2	“Career ladder*”	10,957
S1	“Career progres*”	818

Limiters—Publication Date: 20000101–20231231; Peer Reviewed; English language; Language: English.

Expanders—Apply equivalent subjects.

Search modes—Boolean/Phrase.
